# Bazedoxifene Inhibits Cell Viability, Colony‐Forming Activity, and Cell Migration in Human Non**–**Small Cell Lung Cancer Cells and Improves the Treatment Efficacy of Paclitaxel and Gemcitabine

**DOI:** 10.1111/crj.13822

**Published:** 2024-08-17

**Authors:** Yaochen Huang, Jiayuh Lin, Xiangning Fu, Lequn Li, Shenging Fu

**Affiliations:** ^1^ Department of Thoracic Surgery, Tongji Hospital, Tongji Medical College Huazhong University of Science and Technology Wuhan Hubei China; ^2^ Department of Biochemistry and Molecular Biology, School of Medicine University of Maryland Baltimore USA; ^3^ Laboratory of Thoracic Surgery, Tongji Hospital, Tongji Medical College Huazhong University of Science and Technology Wuhan Hubei China

**Keywords:** bazedoxifene, gemcitabine, IL6, non–small cell lung cancer, paclitaxel, STAT3

## Abstract

**Background:**

Bazedoxifene is a third‐generation selective estrogen receptor modulator that inhibits the IL6/IL6R/GP130 signaling pathway by inhibiting IL6‐induced homodimerization of GP130. Considering that the IL6/IL6R/GP130 signaling pathway is important in tumorigenesis and metastasis, bazedoxifene is thought to have an antitumor effect, which has been proven preliminarily in breast cancer and pancreatic cancer but has not yet been studied in non–small cell lung cancer (NSCLC). This study is aimed at evaluating the antitumor effect of bazedoxifene in NSCLC.

**Methods:**

A549 and H1299 NSCLC cell lines were employed and exposed to various concentrations of bazedoxifene, paclitaxel, gemcitabine, and their combinations for cell viability, colony formation, and wound healing assays to demonstrate the antitumor effect of bazedoxifene with or without paclitaxel or gemcitabine.

**Results:**

MTT cell viability, colony formation, and wound healing assays showed that bazedoxifene was capable of inhibiting cell viability, colony formation, and cell migration in a dose‐dependent manner. In addition, bazedoxifene was capable of working with paclitaxel or gemcitabine synergistically to inhibit cell viability, colony formation, and cell migration.

**Conclusion:**

This study demonstrated the potential antitumor effect of bazedoxifene and its ability to improve the treatment efficacy of paclitaxel and gemcitabine.

## Graphical abstract

Our study showed that bazedoxifene, an FDA‐approved drug, was capable of inhibiting cell viability, colony formation, and cell migration in a dose‐dependent manner and was capable of working with paclitaxel or gemcitabine synergistically. These results demonstrated the potential antitumor effect of bazedoxifene.

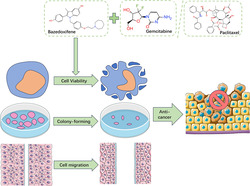

**Abbreviations**
95% CI: 95% confidence intervals
AE: adverse event
ATCC: American Type Culture Collection
CI: combination index
DMEM: Dulbecco's modified Eagle's medium
DMF: N,N‐Dimethylformamide
DMSO: dimethyl sulfoxide
GEO: Gene Expression Omnibus
GP130: Glycoprotein 130
HR: hazard ratio
IC_50_
: half‐maximal inhibitory concentration
IL6: Interleukin 6
IL6R: Interleukin 6 receptor
JAK: Janus kinase
MTT: 3‐(4,5‐Dimethylthiazol‐2‐yl)‐2,5‐diphenyltetrazolium bromide
NSCLC: non–small cell lung cancer
SCLC: small cell lung cancer
STAT3: Signal Transducer and Activator of Transcription 3
TCGA: The Cancer Genome Atlas

## 1 Background

Lung cancer is the second most common cancer worldwide and the most common cancer in China, accounting for 18.4% of all cancer types, with the highest mortality among all cancers [1, 2]. Generally, lung cancers are classified into non–small cell lung cancer (NSCLC) and small cell lung cancer (SCLC). Among them, NSCLC accounts for approximately 80%–85% of all lung cancers [3]. Systemic therapies, including adjuvant chemotherapy, are recommended for Stages II and III and unresectable tumors. Cisplatin‐based two‐drug combinations are preferred for adjuvant chemotherapy, with the second drug being a taxane (such as paclitaxel and docetaxel) or vinca alkaloid (such as gemcitabine and vinorelbine) [4, 5, 6, 7]. However, chemotherapy often causes agony for patients and lowers their compliance because of serious adverse events (AEs) that can sometimes be life‐threatening. Thus, improving drug efficacy and lowering the dose of chemotherapy regimens are important.

The Interleukin 6 (IL6) signaling pathway has proven to be important in tumorigenesis and metastasis. When IL6 interacts with the IL6 receptor (IL6R), IL6 recruits Glycoprotein 130 (GP130), forming the IL6/IL6R/GP130 heterotrimer, which initiates the signaling cascade of phosphorylation of Janus kinases (JAKs) and activates the downstream effector Signal Transducer and Activator of Transcription 3 (STAT3), leading to transcriptional activation. This pathway may be aberrantly regulated and result in malignancy occurrence and progression [8, 9, 10]. Numerous studies have proven that the IL6/GP130/STAT3 signaling pathway plays an important role in NSCLC progression, migration, and invasion via distinct mechanisms [11, 12]. Thus, inhibiting the IL6/GP130/STAT3 signaling pathway may be a promising approach to treating NSCLC.

Bazedoxifene (C_30_H_34_N_2_O_3_) is a third‐generation selective estrogen receptor modulator that has been approved in the United States, the European Union, Japan, and South Korea for the prevention and treatment of menopausal osteoporosis together with conjugated estrogens [13]. Bazedoxifene is relatively safe and well tolerated and hence has promising application prospects [14]. Our previous multiple ligand simultaneous docking and drug repositioning study discovered that bazedoxifene was capable of inhibiting IL6‐induced homodimerization of GP130, hence inhibiting the IL6/GP130/STAT3 pathway [15]. Based on this specific function, we have tried to employ bazedoxifene in treating breast cancer and pancreatic cancer, and the results showed a significant antitumor effect [16, 17, 18]. Since the IL6/GP130/STAT3 signaling pathway also plays an important role in NSCLC, we hypothesized that bazedoxifene may have a similar effect in NSCLC.

This study is aimed at evaluating the inhibitory effect of bazedoxifene on cell viability, colony‐forming activity, and cell migration and its ability to improve the treatment efficacy of paclitaxel and gemcitabine in NSCLC.

## 2 Materials and Methods

### 2.1 Cell Lines and Reagents

The human NSCLC cell lines A549 and H1299 were acquired from the American Type Culture Collection (ATCC; Manassas, VA, United States). All cell lines were cultured in Dulbecco's modified Eagle's medium (DMEM; Mediatech Inc., Manassas, VA, United States) routinely with 4.5 g/L L‐glutamine and sodium pyruvate supplemented with 10% fetal bovine serum (FBS; Atlanta Biologicals, Flowery Branch, GA, United States) and 1% penicillin/streptomycin (Sigma‐Aldrich, Merck KGaA, Darmstadt, Germany) in a humidified 37°C incubator with 5% CO_2_. Cells were observed under the microscope routinely to assess the expected morphology.

The reagents used in this study were as follows: bazedoxifene acetate (Sigma‐Aldrich, Merck KGaA), paclitaxel (LC Laboratories, Woburn, MA), gemcitabine (Sigma‐Aldrich, Merck KGaA), dimethyl sulfoxide (DMSO; Sigma‐Aldrich, Merck KGaA), 3‐(4,5‐dimethylthiazol‐2‐yl)‐2,5‐diphenyltetrazolium bromide (MTT; Sigma‐Aldrich; Merck KGaA), crystal violet (Sigma‐Aldrich, Merck KGaA), and N,N‐dimethylformamide (DMF; Fisher Scientific, Waltham, MA, United States). Bazedoxifene, paclitaxel, and gemcitabine were dissolved in sterile DMSO to make 20 mM stock solutions, and they were stored at −20°C according to the instructions of the manufacturers.

### 2.2 MTT Cell Viability Assay

A549 and H1299 NSCLC cells were seeded in 96‐well microtiter plates at a density of 3000 cells per well and grown overnight at 37°C in 100 μL DMEM with 10% FBS. The following day, the culture medium in each well was replaced with 100 μL DMEM and 10% FBS supplemented with various concentrations of bazedoxifene (1, 2.5, 5, 10, 20, and 40 μM), paclitaxel (0.001, 0.005, 0.01, 0.05, 0.1, and 0.5 μM), gemcitabine (0.1, 0.5, 1, 5, 10, and 20 μM), the combination of bazedoxifene (2.5 μM for the A549 cell line and 5 μM for the H1299 cell line) with paclitaxel (0.001 μM for both cell lines) or gemcitabine (0.5 μM for both cell lines), and DMSO control at 37°C. After a 72‐h incubation, 25 μL of MTT solution was added to each well, and the cells were incubated for 4 h. Afterward, 150 μL of DMF solubilization solution was added to each well, the plates were placed on a shaker, and the cells were incubated overnight at room temperature in a sealed, moistened, lightless chamber. Cell viability was quantified by measuring the absorbance at 595 nm in each well compared with the absorbance in the control wells. Half‐maximal inhibitory concentrations (IC_50_) were determined using SigmaPlot 9.0 software (Systat Software, Inc., San Jose, CA, United States).

### 2.3 Colony Formation Assay

The culture period is 11 days. Firstly, A549 and H1299 cells were seeded in six‐well plates at a density of 500 cells per well and grown overnight at 37°C. The following day, the cells were incubated with various concentrations of bazedoxifene (1, 2.5, 5, and 10 μM), paclitaxel (0.001, 0.005, 0.01, 0.05, and 0.1 μM), gemcitabine (0.01, 0.05, 0.1, 0.5, and 1 μM), the combination of bazedoxifene (2.5 μM for the A549 cell line and 2 μM for the H1299 cell line) with paclitaxel (0.001 or 0.005 μM for both cell lines) or gemcitabine (0.01 or 0.02 μM for the A549 cell line and 0.001, 0.005, or 0.025 μM for the H1299 cell line), and DMSO. The medium in each well was changed to a fresh medium without drugs after incubation for 72 h. One week later, the cells were washed twice with PBS, fixed with cold methanol for 30 min at 4°C, and stained with 1% crystal violet dye (dissolved in 25% methanol) at room temperature for 1 h. The plates were washed with distilled water, dried, and scanned for analysis. The number of cell colonies was counted by three independent observers with the help of Fiji software [19].

### 2.4 Wound Healing Assay for Cell Migration

A549 and H1299 cells were seeded in six‐well plates and incubated at 37°C overnight. When the cells reached 100% confluence, the monolayer was scratched to create a wound using a 100‐μL pipette tip. The monolayer was washed with sterilized PBS, and the cells were treated with various concentrations of bazedoxifene (1, 2.5, 5, and 10 μM), paclitaxel (0.001, 0.005, and 0.01 μM for the A549 cell line and 0.01, 0.05, and 0.1 μM for the H1299 cell line), gemcitabine (0.001, 0.005, 0.01, and 0.05 μM for both cell lines), the combination of bazedoxifene (2.5 μM for both cell lines) with paclitaxel (0.001 or 0.005 μM for the A549 cell line and 0.01 or 0.05 μM for the H1299 cell line) or gemcitabine (0.01 or 0.05 μM for both cell lines), and DMSO, incubated at 37°C; images were captured with a Nikon Eclipse TS100 microscope at 0 h, 15 h (for the H1299 cell line), and 23 h (for the A549 cell line). The width of the scratch line was quantified by three independent observers with the help of Fiji software, and the measurements were employed as an indicator of cell migration [19]. Relative wound healing ability was calculated using the following formula: percent wound healing = [(width at 0 h − width at 15 or 23 h)/(width at 0 h)] × 100; the average was calculated from five replicates [16].

### 2.5 Statistical Analysis

The combination index (CI) is a quantitative determination of drug interactions based on an extension of the general equation for the single drug effect. The general equation for CI calculation is 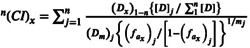, where 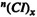 is the CI at *x*% inhibition for *n* drugs, ${\left(D_{x}\right)}_{1-n}$ is the sum of the dose of *n* drugs that results in *x*% inhibition in combination, ${\left[D\right]}_{j}/\sum_{1}^{n}\left[D\right]$ is the proportionality of the dose of each of n drugs that results in *x*% inhibition in combination, and ${\left(D_{m}\right)}_{j}{\left\{{\left(f_{a_{x}}\right)}_{j}/\left[1-{\left(f_{a_{x}}\right)}_{j}\right]\right\}}^{1/m_{j}}$ is the dose of each drug alone that results in *x*% inhibition, where *Dm* is the median‐effect dose, $f_{a_{x}}$ is the fractional inhibition at *x*% inhibition, and *m* is the slope of the median‐effect plot portraying the shape of the dose‐effect curve [20]. CI was established using data obtained from the MTT, colony formation, and wound healing assays with CompuSyn software (http://www.combosyn.com). CI values indicate a synergistic effect when < 1, an antagonistic effect when > 1, and an additive effect when equal to 1. Other statistical analyses and plotting were carried out using GraphPad Prism 5 (GraphPad Software Inc., San Diego, CA, United States) software and the “ggplot2” packages of R 4.2.1. Differences between groups were analyzed by Student's *t*‐test. The data are presented as the means and standard error of the mean. The significance was set at *p* < 0.05. *, **, and *** indicate *p* < 0.05, *p* < 0.01, and *p* < 0.001, respectively.

## 3 Results

### 3.1 Bazedoxifene Inhibited NSCLC Cell Viability and Worked With Paclitaxel or Gemcitabine Synergistically

**FIGURE 1:**
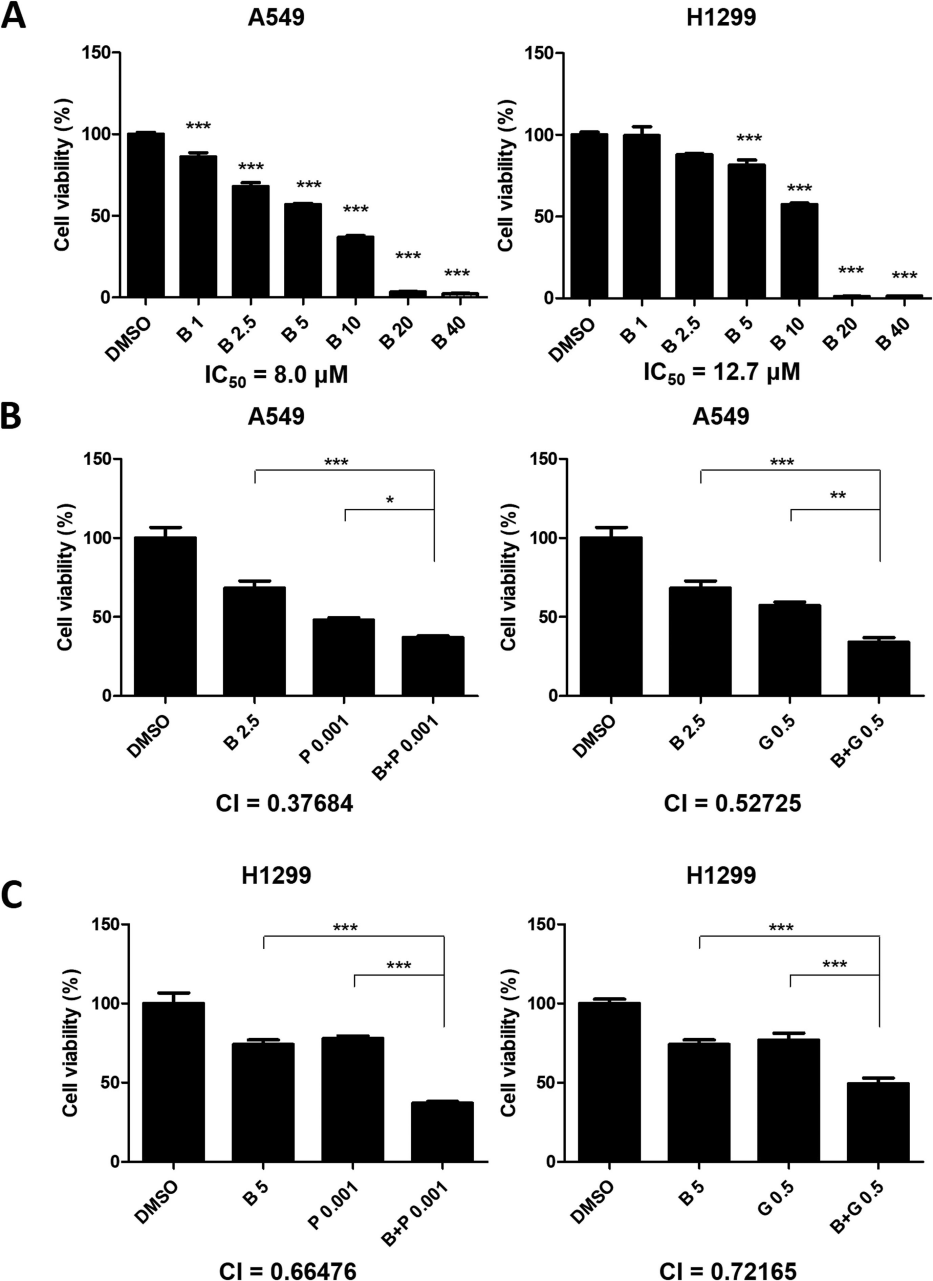
Histograms showing the viability of NSCLC cells exposed to different drugs and incubated for 72 h demonstrated that bazedoxifene (B) inhibited the cell viability of non–small cell lung cancer (NSCLC) cells in a dose‐dependent manner. Bazedoxifene combined with paclitaxel (P) or gemcitabine (G) synergistically inhibited the cell viability of NSCLC cells. (A) A549 and H1299 cells were exposed to various concentrations of bazedoxifene, which showed a dose‐dependent inhibition effect on cell viability. (B) A549 cells were exposed to DMSO, 2.5 μM bazedoxifene, 0.001 μM paclitaxel, 0.5 μM gemcitabine, and 2.5 μM bazedoxifene plus 0.001 μM paclitaxel or 0.5 μM gemcitabine. CI values were calculated using data obtained from the different drug treatments. (C) H1299 cells were exposed to DMSO, 5 μM bazedoxifene, 0.001 μM paclitaxel, 0.5 μM gemcitabine, and 5 μM bazedoxifene plus 0.001 μM paclitaxel or 0.5 μM gemcitabine.

To explore the ability of bazedoxifene to inhibit the viability of NSCLC cells, we performed MTT assays. After being exposed to different concentrations of bazedoxifene (1, 2.5, 5, 10, 20, and 40 μM) and incubated for 72 h, cell viability decreased to different extents, as shown in Figure 1A. The viability of both A549 and H1299 cells was significantly reduced by bazedoxifene, and the effect was dose‐dependent (for A549 cells, *p* < 0.001 for all comparisons; for H1299 cells, *p* = 0.084 and *p* = 0.053 for 1 and 2.5 μM, respectively, and *p* < 0.001 for the rest). The IC_50_ for A549 and H1299 cells were 8.0 μM and 12.7 μM, respectively.

To explore the synergistic effect of bazedoxifene combined with paclitaxel or gemcitabine, A549 and H1299 cells were exposed to bazedoxifene, paclitaxel, gemcitabine, the combination of bazedoxifene plus paclitaxel, and the combination of bazedoxifene plus gemcitabine. For the A549 cell line, both the bazedoxifene (2.5 μM) plus paclitaxel (0.001 μM) and bazedoxifene (2.5 μM) plus gemcitabine (0.5 μM) groups showed a significantly stronger inhibitory effect on cell viability than each drug alone after incubation for 72 h (*p* < 0.001, *p* = 0.032, *p* < 0.001, and *p* = 0.0078, respectively), as shown in Figure 1B. The CI values were both less than 1 (0.37684 for the bazedoxifene plus paclitaxel group and 0.52725 for the bazedoxifene plus gemcitabine group), suggesting a synergistic effect. For the H1299 cell line, bazedoxifene (5 μM) plus paclitaxel (0.001 μM) or gemcitabine (0.05 μM) exhibited a better inhibitory effect on cell viability than each drug alone (*p* < 0.001 for all groups), as presented in Figure 1C. The CI values were both less than 1 (0.66476 for the bazedoxifene plus paclitaxel group and 0.72165 for the bazedoxifene plus gemcitabine group), indicating a synergistic effect.

### 3.2 Bazedoxifene Inhibited the Colony Formation Capability of NSCLC Cells and Worked With Paclitaxel or Gemcitabine Synergistically

**FIGURE 2:**
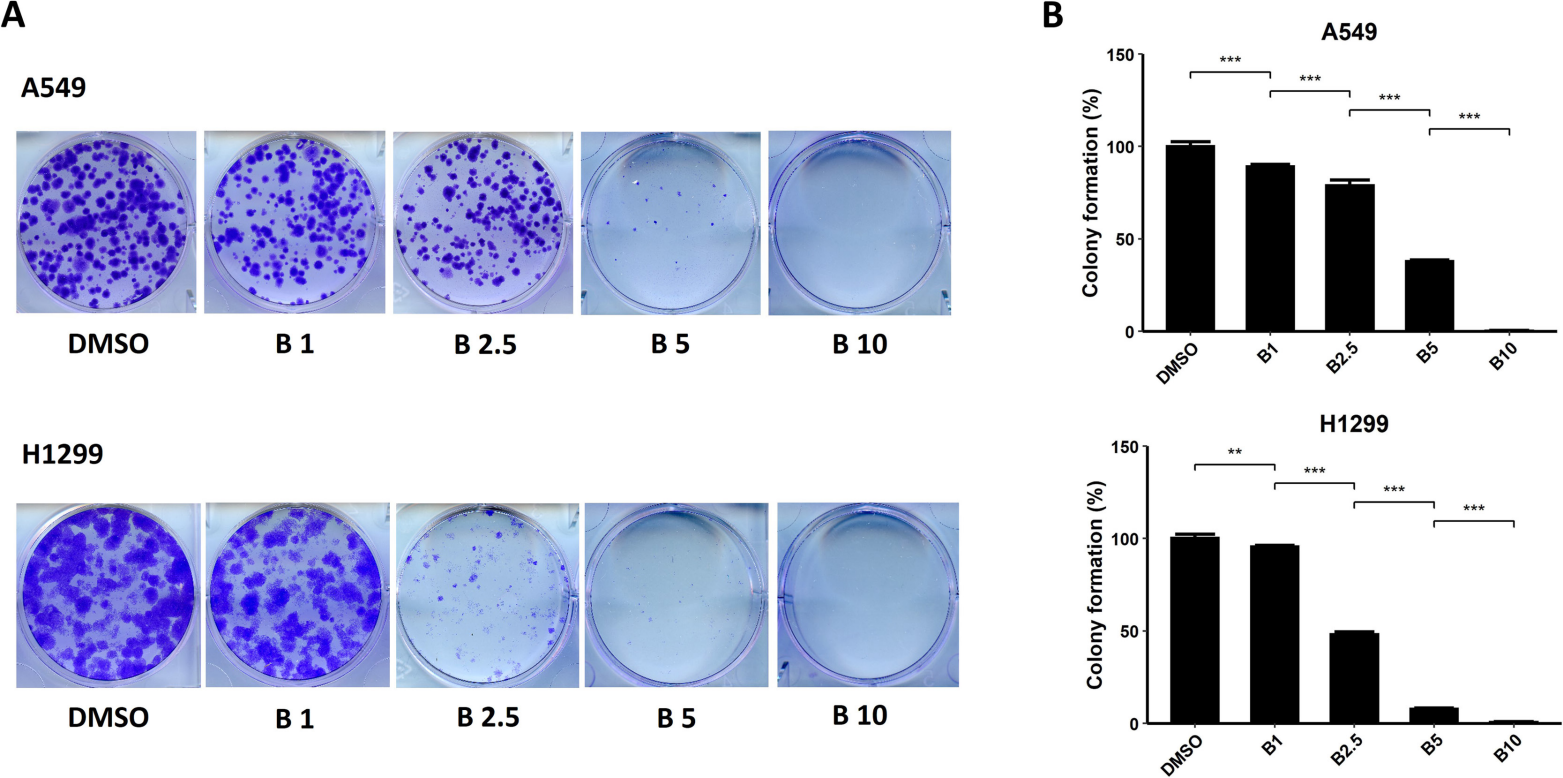
Colony formation assays demonstrated that bazedoxifene (B) inhibited the colony‐forming activity of non–small cell lung cancer (NSCLC) cells dose‐dependently. (A) Photos of A549 and H1299 cells captured after incubation for a week in DMSO or various concentrations of bazedoxifene. (B) Histograms of the percentages of colony formation calculated from colony counting of the images in A. The results showed that colony formation activity was inhibited significantly in a dose‐dependent manner.

To explore the capability of bazedoxifene to inhibit colony formation in NSCLC cells, we performed colony formation assays. After incubation in different concentrations of bazedoxifene (1, 2.5, 5, and 10 μM) for a week, the number of colonies formed decreased with increasing concentration in both A549 and H1299 cells, as presented in Figure 2A. A quantitative analysis of colony numbers was conducted, and the results are shown in Figure 2B. The results showed that the colony formation inhibition effect acted in a dose‐dependent manner, which was in agreement with the results from the cell viability assays (for A549 cells, *p* < 0.001 for all comparisons; for H1299 cells, *p* = 0.0029 for 1 μM and *p* < 0.001 for the rest).

**FIGURE 3:**
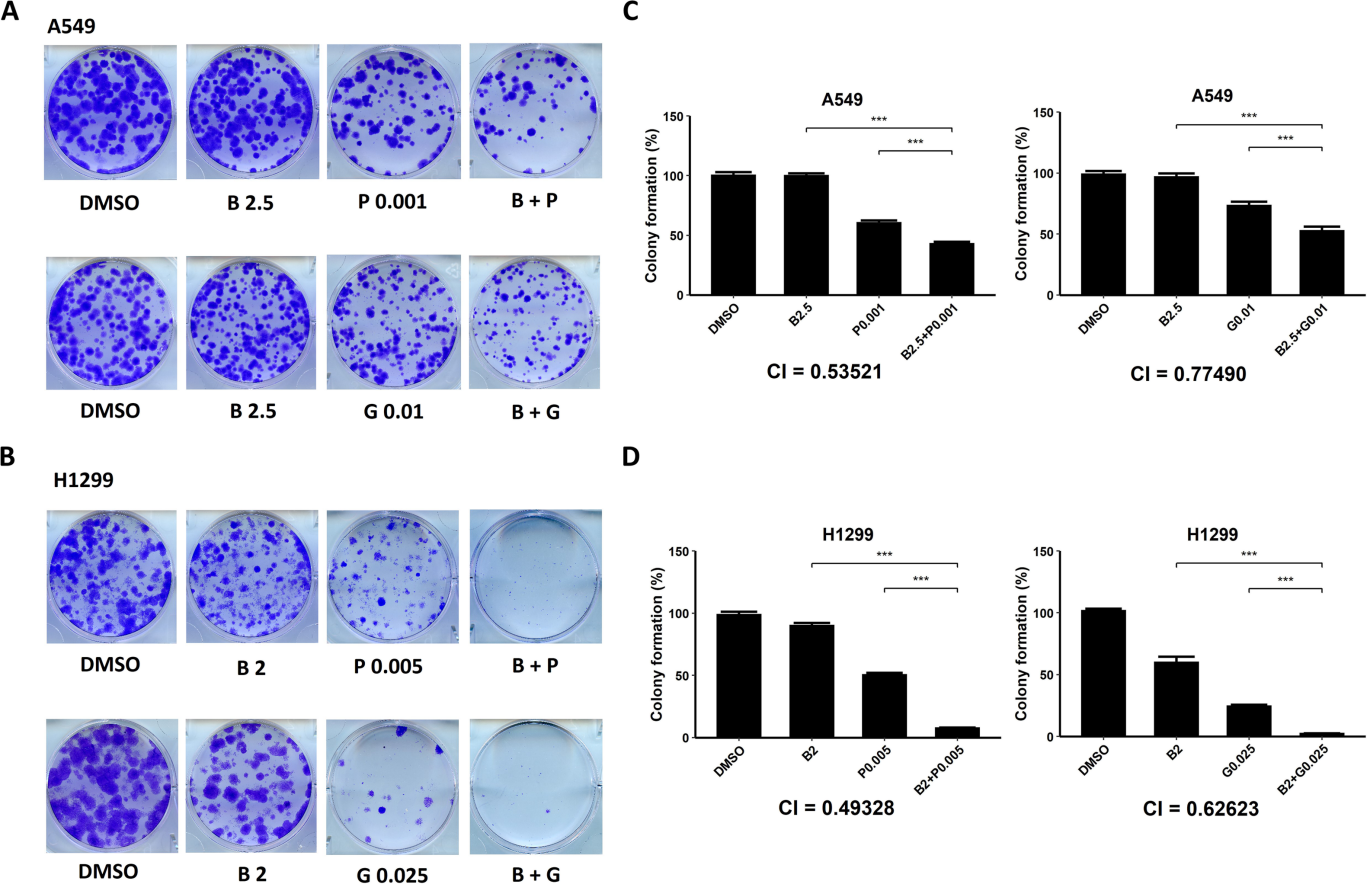
The combination of bazedoxifene (B) and paclitaxel (P) or gemcitabine (G) showed a stronger inhibition effect on the colony‐forming activity of non–small cell lung cancer (NSCLC), suggesting that bazedoxifene worked synergistically with paclitaxel or gemcitabine. (A) Photos of A549 cells captured after incubation for a week in DMSO, 2.5 μM bazedoxifene (B), 0.001 μM paclitaxel (P), 0.01 μM gemcitabine (G), and the combination of 2.5 μM bazedoxifene with 0.001 μM paclitaxel or 0.01 μM gemcitabine. (B) Photos of H1299 cells captured after incubation for a week in DMSO, 2 μM bazedoxifene, 0.005 μM paclitaxel, 0.025 μM gemcitabine, and the combination of bazedoxifene with 0.005 μM paclitaxel or 0.025 μM gemcitabine. (C) Histograms of the percentages of colony formation of A549 cells calculated from colony counting of the images in A. CI values were calculated using data obtained from the different drug treatments. (D) Histograms of the percentages of the colony formation of A549 cells calculated from the colony counting of the images in B.

Next, we investigated the combination of bazedoxifene with paclitaxel or gemcitabine. As presented in Figure 3A, for the A549 cell line, the combination of bazedoxifene (2.5 μM) and paclitaxel (0.001 or 0.005 μM) or gemcitabine (0.01 or 0.02 μM) exhibited a better colony formation inhibition effect than each drug alone. For the H1299 cell line, the colony‐forming activity was significantly lower in the combination of bazedoxifene (2 μM) and paclitaxel (0.001 or 0.005 μM) or gemcitabine (0.001, 0.005, or 0.025 μM) groups than in each monotherapy, as shown in Figure 3B. The results of the quantitative analysis of colony numbers are shown in Figure 3C,D (*p* < 0.001 for all groups). CI values were calculated, and the results were all less than 1 (A549 cell line, 0.53521 for bazedoxifene plus paclitaxel and 0.77490 for bazedoxifene plus gemcitabine; H1299 cell line, 0.49328 for bazedoxifene plus paclitaxel and 0.62623 for bazedoxifene plus gemcitabine), suggesting a synergistic effect, which was consistent with the results from the cell viability assays.

### 3.3 Bazedoxifene Inhibited NSCLC Cell Migration Synergistically With Paclitaxel or Gemcitabine

**FIGURE 4:**
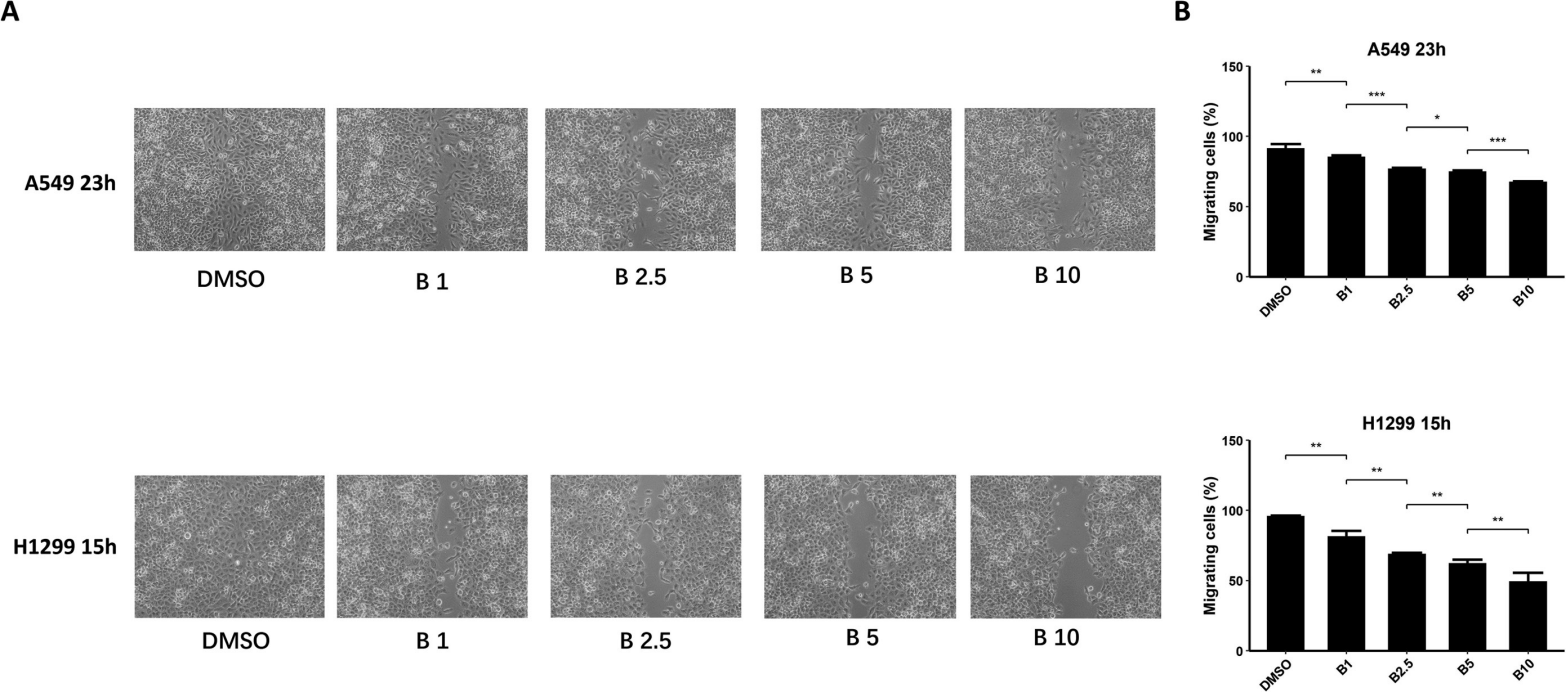
Cell migration demonstrated that bazedoxifene (B) inhibited cell migration of non–small cell lung cancer (NSCLC) in a dose‐dependent manner. (A) Photos of A549 and H1299 cells captured under the microscope after they were scratched and then incubated in DMSO or various concentrations of bazedoxifene (B) for 23 h (A549 cells) or 15 h (H1299 cells). (B) Histograms of the percentage of migrating cells from A measured and calculated by researchers.

To explore the ability of bazedoxifene to inhibit NSCLC cell migration, we performed wound healing assays to evaluate cell migration. A549 and H1299 cells were incubated for 23 h and 15 h, respectively, after being scratched. As demonstrated in Figure 4A,B, the pictures captured and quantitative analysis of the scratch width after exposure to various concentrations of bazedoxifene (1, 2.5, 5, and 10 μM) showed that the migratory potential was reduced significantly in a dose‐independent manner, which was consistent with the results from the cell viability assays and colony formation assays (for A549 cells, *p* = 0.0099, *p* < 0.001, *p* = 0.02, and *p* < 0.001, respectively; for H1299 cells, *p* = 0.0013, *p* = 0.002, *p* = 0.005, and *p* = 0.0073, respectively).

**FIGURE 5:**
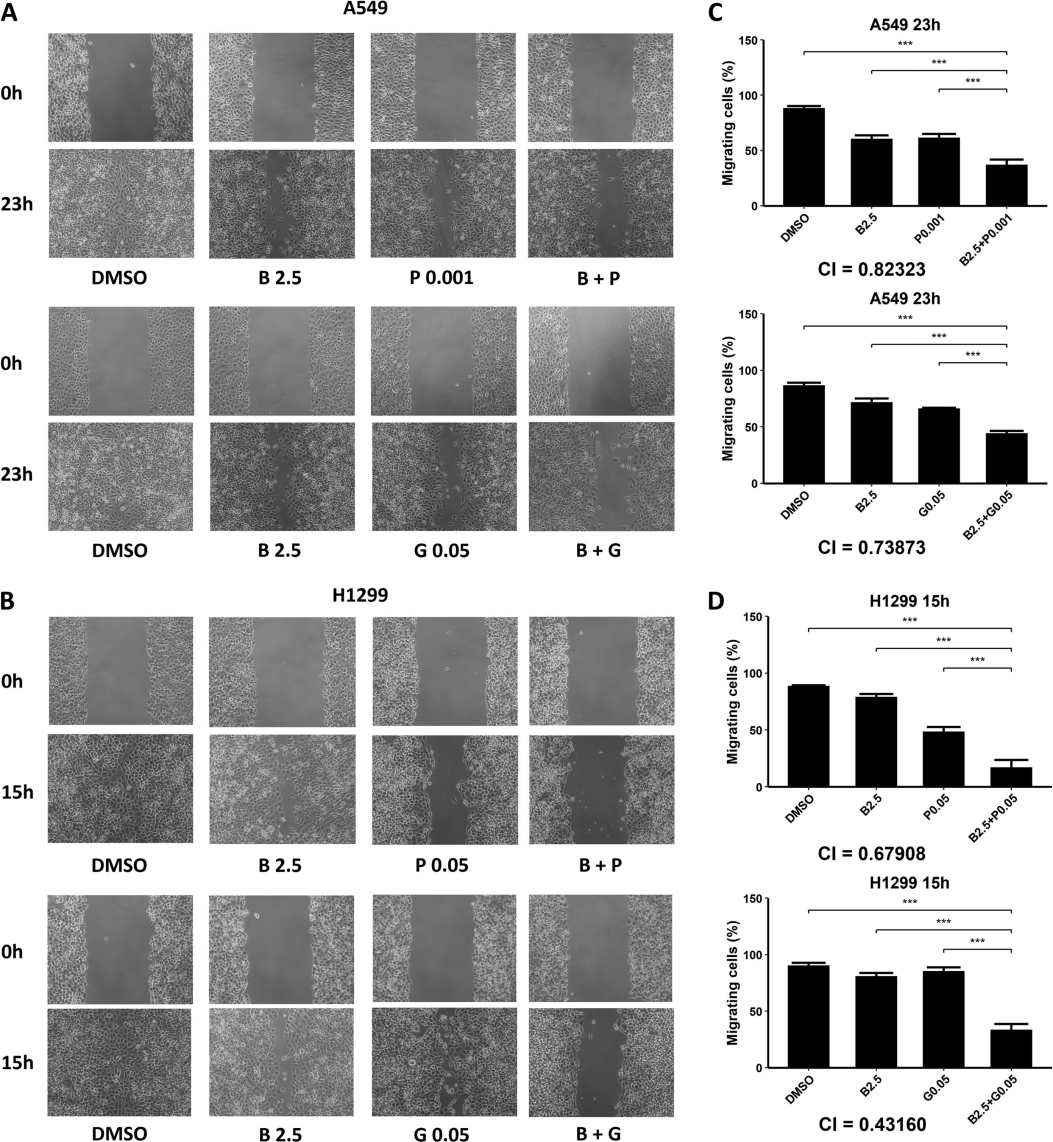
Cell migration showed that combined treatment of bazedoxifene (B) and paclitaxel (P) or gemcitabine (G) inhibited cell migration of non–small cell lung cancer (NSCLC) synergistically. (A) Photos of A549 cells captured under a microscope after they were scratched and then incubated for 23 h in DMSO, 2.5 μM bazedoxifene, 0.001 μM paclitaxel, 0.05 μM gemcitabine, and the combination of bazedoxifene with paclitaxel or gemcitabine. (B) Photos of H1299 cells captured under the microscope after they were scratched and then incubated for 15 h in DMSO, 2.5 μM bazedoxifene, 0.05 μM paclitaxel, 0.05 μM gemcitabine, and the combination of bazedoxifene with paclitaxel or gemcitabine. (C) Histograms of the percentage of migrating A549 cells from A. (D) Histograms of the percentage of migrating H1299 cells from B were measured and calculated by researchers.

We next exposed A549 and H1299 cells to bazedoxifene (2.5 μM for both cell lines), paclitaxel (0.001 or 0.005 μM for the A549 cell line and 0.01 or 0.05 μM for the H1299 cell line), gemcitabine (0.01 or 0.05 μM for both cell lines), or their combinations. The pictures captured before and after incubation are shown in Figure 5A,B, and matched quantitative analysis results are shown in Figure 5C,D. The results showed that the combination of bazedoxifene and paclitaxel or gemcitabine inhibited cell migration more than each monotherapy (*p* < 0.001 for all groups). CI values were calculated and were all less than 1 (A549 cell line, 0.82323 for bazedoxifene plus paclitaxel and 0.73873 for bazedoxifene plus gemcitabine; H1299 cell line, 0.67908 for bazedoxifene plus paclitaxel and 0.43160 for bazedoxifene plus gemcitabine), suggesting a synergistic effect, which was consistent with the results from the cell viability assays and colony formation assays.

## 4 Discussion

As a third‐generation selective estrogen receptor modulator, bazedoxifene has been proven to inhibit tumor cell growth and migration by inhibiting the IL6/GP130 signaling pathway. This effect has been presented in several types of cancer, including breast cancer and pancreatic cancer [17, 18]. No prior studies have explored this effect in NSCLC so far, and our study demonstrated for the first time that bazedoxifene had a dose‐dependent inhibition effect and that this effect was synergistic when it was combined with paclitaxel or gemcitabine on cell viability, colony formation, and cell migration of A549 and H1299 NSCLC cells in vitro via a series of assays, including MTT cell viability assays, colony formation assays, and wound healing assays.

Evidences show that the expression of IL6 is significantly higher in NSCLC tissues, and a high expression level of IL6 exhibits the potential to predict the occurrence and metastasis of lung cancer [21, 22]. Other studies note that STAT3 was highly expressed in over 50% of NSCLCs, and an increased STAT3 expression level acted as a poor prognosis biomarker, predicting poor differentiation of cancer cells, lymph node metastasis, a low survival rate, and drug resistance [23, 24, 25, 26]. The aberrant activation of the IL6/GP130/STAT3 signaling pathway and the IL6 feed‐forward loop formation are the drivers of tumorigenesis in many types of cancer, including NSCLC [9]. When the expression of IL6 increases aberrantly, the phosphorylation of STAT3 will be promoted, resulting in transcriptional activation. Increased levels of phosphorylated STAT3 will promote the autocrine of IL‐6 and GP130, hence forming a feed‐forward loop; accelerating transcriptional activation; and leading to tumorigenesis, invasiveness, metastasis, and drug resistance [9, 26, 27].

Considering the importance of the IL6/GP130/STAT3 signaling pathway in tumorigenesis and progression, targeting this pathway is a promising approach to cancer treatment. Targeted inhibitors of the key factors of the IL6 signaling pathway, including IL6 (such as siltuximab), IL6R (such as tocilizumab), GP130 (such as olamkicept), and STAT3 (such as danvatirsen), have been widely studied, and many drugs have been employed in clinical trials for treating some types of cancers [10]. However, there are few results of large‐scale clinical randomized controlled trials for NSCLC targeting the IL6/GP130/STAT3 signaling pathway that have been published. In addition, some of the new drugs have been shown to have some lethal adverse effects, such as rapidly induced thrombocytopenia when employing danvatirsen for cancer treatment, which could reduce the value of their application [28]. Developing novel drugs requires many efforts and expenditures, and unapproved drugs require rigorous trials to test their efficacy and safety, which also require a very long time and many expenditures to accomplish. Thus, studying approved drugs is advantageous.

Our previous study has shown that bazedoxifene is capable of blocking the IL6/GP130/STAT3 signaling pathway by inhibiting IL6‐induced homodimerization of GP130, leading to a potential antitumor effect, which has been demonstrated in triple‐negative breast cancer and pancreatic cancer [15, 16, 17]. As an FDA‐approved drug, bazedoxifene is superior in regards to its safety and tolerance, with a similar serious AE incidence rate compared to placebo and the most frequent AEs being back pain and arthralgia [14]. However, for the classic chemotherapy drugs paclitaxel and gemcitabine, the AEs are obvious, with the most frequent AE being hemopathy, which is life‐threatening and unbearable [29]. Since this toxicity is dose‐limiting, lowering the dosage will effectively decrease the incidence of AEs. As our study demonstrated, bazedoxifene was capable of working with paclitaxel or gemcitabine synergistically. Hence, it is promising to employ bazedoxifene combined with paclitaxel or gemcitabine for cancer treatment to lower the dosage of paclitaxel or gemcitabine and reduce associated AEs without increasing risk.

Our study showed the potential application value of bazedoxifene as an antitumor drug. However, the efficacy and safety of bazedoxifene working as an antitumor drug have not been testified in humans yet, which requires clinical trials to provide stronger evidences. Moreover, our study is limited to the cell level, lacking evidences at the molecular level to elucidate the mechanism within, which requires further researches to explore.

## 5 Conclusion

This study demonstrated the potential of bazedoxifene as an antitumor drug and the capability of bazedoxifene to work synergistically with paclitaxel or gemcitabine to lower the effective dosage of chemotherapy regimens in NSCLC by MTT cell viability assays, colony formation assays, and wound healing assays.

## Author Contributions

Y.H., S.F., X.F., L.L., and J.L. designed the study. Y.H., S.F., and L.L. performed the experiments and collected the data. Y.H., S.F., X.F., and J.L. contributed to writing the manuscript. Y.H., S.F., and X.F. participated in the data analyses. All authors contributed to the article and approved the submitted manuscript.

## Ethics Statement

The authors have nothing to report.

## Consent

The authors have nothing to report.

## Conflicts of Interest

The authors declare no conflicts of interest.

## Data Availability

The data that support the findings of this study are available from the corresponding author upon reasonable request.
